# Development of a 24-hour movement index: exploring acceptability among Canadian parents

**DOI:** 10.3389/fspor.2025.1571207

**Published:** 2025-07-30

**Authors:** Jian Kun Zhan, Karim M. Khan, Mark S. Tremblay, Guy Faulkner

**Affiliations:** ^1^ School of Kinesiology, University of British Columbia, Vancouver, BC, Canada; ^2^ Department of Family Practice, Faculty of Medicine, University of British Columbia, Vancouver, BC, Canada; ^3^ Healthy Active Living and Obesity Research Group, Children’s Hospital of Eastern Ontario Research Institute, Ottawa, ON, Canada; ^4^ Department of Health Sciences, Carleton University, Ottawa, ON, Canada; ^5^ Department of Pediatrics, University of Ottawa, Ottawa, ON, Canada

**Keywords:** movement behaviours, guidelines, knowledge translation, acceptability, monitoring, mobile health

## Abstract

**Background:**

The Canadian 24-Hour Movement Guidelines for Children and Youth were introduced in 2016. They offer recommendations on moderate-to-vigorous physical activity, light physical activity, sedentary behaviour, and sleep in a typical 24-hour period to achieve optimal health outcomes. However, a lack of awareness and knowledge about the guidelines among children and parents is a concerning public health issue and may contribute to the low guideline adherence of Canadian children. A “Movement Index” app is planned to help parents track their children's movement behaviours through manual data entry and/or a wearable device. The Movement Index would also demonstrate to parents how the combination of their children's movement behaviours, such as a change in time reallocation, may be associated with different health outcomes. Using the Theoretical Framework of Acceptability, the objectives of this study were to (1) explore interest in, and acceptability of, the proposed Movement Index, and (2) identify potential refinements in developing the app.

**Methods:**

Individual semi-structured interviews were conducted over Zoom with 22 parents of children aged 5–11 years from across Canada. Interview data were analyzed with thematic analysis using a constant comparative method.

**Results:**

Results suggest that the Movement Index is acceptable on two constructs (perceived effectiveness, intervention coherence), mostly acceptable on two (affective attitude, ethicality), and has mixed acceptability for the remaining three (burden, opportunity cost, self-efficacy).

**Discussion:**

On balance, the Movement Index was found to be acceptable, and the project should proceed with several caveats that need to be addressed regarding accessibility and ethical concerns. Future work is required to develop and pilot the Movement Index before further re-examining its acceptability and usability.

## 1 Introduction

Childhood exposure to healthy movement behaviours provides a window for developing healthy developmental trajectories (1). While many previous studies have emphasized the separate health benefits of achieving adequate physical activity, sufficient sleep, and limiting time spent sedentary (2, 3, 4), the Canadian 24-Hour Movement Guidelines for Children and Youth introduced in 2016 (24HMG; 5) now integrate recommendations for all these behaviours. These guidelines offer recommendations for children and youth aged 5–17 years regarding moderate-to-vigorous physical activity (MVPA), light physical activity, sedentary behaviour, and sleep in a typical 24-h period. This new integrated conceptualization of movement behaviours emphasizes the holistic inter-relationship of all movement behaviours throughout the day, with the reallocation of time from sedentary behaviour to MVPA while protecting sleep time associated with optimal health outcomes (5). Reviews consistently demonstrate mental and physical health benefits from achieving healthy amounts of all three behaviors (6).

Adherence to 24-h movement guidelines during early life promotes a number of benefits in adulthood, including lower rates of hypertension (7), obesity (8), type 2 diabetes (9), and midlife mortality (10). Despite clear evidence that meeting guideline recommendations and achieving a healthy balance of movement behaviours is associated with improved physical and mental health, most children and youth fail to meet the three 24-h movement guidelines, particularly adolescents, girls, and those who are from countries with a lower Human Development Index (11). In 2024, an average of only 4% of children and youth in Canada were meeting the physical activity, screen time and sleep duration recommendations within the Canadian 24HMG (12). One challenge may be the lack of awareness of the guidelines in the first place with one evidence synthesis showing that Canadian guideline awareness was low among mothers, the general population, early childhood education students, and pediatricians (13). In a more recent cross-sectional online survey with parents/guardians (*n* = 576) of young children in Canada, few participants (11.9%) reported being familiar with the 24HMG (14). From one theoretical perspective (Hierarchy of Effects Model, 15), awareness and understanding are causally linked to distal outcomes (e.g., behaviour change) through a series of intermediate changes (e.g., attitudes, self-efficacy, intentions). Thus, low public adherence to guidelines might be explained in part by the low public awareness of those guidelines. This may result from inadequate dissemination of the guidelines (13, 16). Therefore, dissemination strategies for the 24HMG in the general population are needed to increase uptake and adoption (17).

Given the population reach of mobile technology in Canada (18, 19), utilizing mobile health to promote awareness of the 24HMG is an intuitively appealing strategy. Mobile health can provide features such as personalized feedback, progress tracking, and self-monitoring which may increase compliance with guidelines (20). A recent systematic review showed that mobile health app-based interventions could be effective in increasing physical activity and improving fitness in children and adolescents (21). Since children and youth spend a significant portion of their time under the care of their parents or legal guardians, such mobile health initiatives could focus on caretakers and family members in supporting healthy movement behaviours of children at a population level (22). These issues have sparked interest in developing a mobile health app, currently referred to as the “Movement Index”, that is based on a single, individualized metric that considers daily physical activity, sedentary behaviour, and sleep. The Movement Index could help parents monitor their children's movement behaviours, and demonstrate how a change in time reallocation (e.g., spending less time sitting and doing more physical activity) is associated with changes in mental and physical health outcomes. Epidemiologic research with large longitudinal datasets to explore and develop the evidence underpinning the Movement Index algorithm is still a work in progress and is far from complete. However, it is hoped that the Movement Index may serve as a knowledge translation tool for the public and increase awareness and understanding of the 24HMG, and how to integrate these guidelines into a family's everyday life for improved health and wellness. In light of limited knowledge translation efforts to support the release of the 24HMG for Children and Youth (23), any new tool such as the proposed Movement Index would be a beneficial addition to health promotion efforts in Canada.

When developing new guidelines or interventions, it is critical to engage stakeholders and end-users to ensure that what is being developed matches their needs (24). Before further investing in creating the Movement Index, it is important to examine whether this innovative tool is perceived as acceptable in a parent's daily life. The Theoretical Framework of Acceptability (TFA) (25) offers an evidence-based and multi-construct theoretical framework of acceptability to assess how potential users perceive a new intervention to increase potential uptake. This framework consists of seven constructs that capture the different dimensions of acceptability (see Table 1). As part of the framework, acceptability can be assessed from two temporal perspectives (prospective and retrospective) and from three different time points in relation to the intervention delivery period (pre-intervention, during intervention, and post-intervention). For the current study, acceptability was assessed from a prospective temporal perspective at the pre-intervention time period. Findings from previous qualitative studies on health interventions using the TFA (26, 27) suggest that it is an appropriate framework to deductively assess the acceptability of a health intervention. This formative research using the TFA is an important step in ensuring that the Movement Index is aligned with the interests and values of parents in Canada.

**Table 1:** Theoretical framework of acceptability (16).

Acceptability is a multi-faceted construct that reflects the extent to which people delivering or receiving a healthcare intervention consider it to be appropriate, based on anticipated or experienced cognitive and emotional responses to the intervention.	
Construct of acceptability	Construct definition
Intervention coherence	The extent to which the participant understands the intervention and how it works
Perceived effectiveness	The extent to which the intervention is perceived as likely to achieve its purpose
Affective attitude	How an individual feels about the intervention
Burden	The perceived amount of effort that is required to participate in the intervention
Opportunity cost	The extent to which benefits, profits or values must be given up by engaging in the intervention
Self-efficacy	The participant's confidence that they can perform the behaviour(s) required to participate in the intervention
Ethicality	The extent to which the intervention has good fit with an individual's value system

## 2 Methods and methodology

### 2.1 Recruitment and sampling

Recruitment began following institution ethics approval by the University of British Columbia's Research Ethics Board (# H22-02678). Parents (*n* = 22) were recruited in December 2023 via an invitation to existing users of the ParticipACTION app. ParticipACTION is a Canadian non-profit, physical activity social marketing organization. The organization created an app to promote physical activity and educate the Canadian population on the benefits of regular physical activity (28). Interested participants self-identified and contacted the research team via email. The participants were eligible to participate if they resided in Canada, spoke English to ensure interview comprehension, and were parents of children aged 5–11 years as the Movement Index is currently conceptualized as a tool for parents to monitor their children's, and not youth, movement behaviors. No formal data saturation criterion was established for this study. The sample size was determined based on feasibility and was deemed sufficient to provide rich, diverse insights for thematic analysis. The demographic profile of the sample is presented in Table 2. The sample consisted of 11 participants identifying as mothers and 11 as fathers, with the majority identifying as white, married, well-educated, employed full-time, and from British Columbia. While this sample is mostly representative of the Canadian parent population, there is an overrepresentation of educated and active individuals.

**Table 2:** Demographic information of participants.

Demographic Characteristic	Participants
Participants	Total: 22
Gender	Father (F): 11 Mother (M): 11
Age group (years)	25–34: 4 35–44: 8 45–54: 8 55+: 2
Ancestry	White: 15 East Asian: 5 Black: 1 Indigenous: 1
Marital Status	Married: 19 Widowed: 1 Never Married: 1 Divorced: 1
Education	Bachelors: 11 Graduate: 7 College: 3 High School: 1
Employment	Full time: 19 Part time: 3
Location	British Columbia: 9 Ontario: 5 Manitoba: 4 Alberta: 2 Saskatchewan: 1 Newfoundland and Labrador: 1
Days of Physical Activity per Week	3.59 ± 1.76
Number of Children	2.18 ± 1.01

### 2.2 Data collection

**Figure 1:**
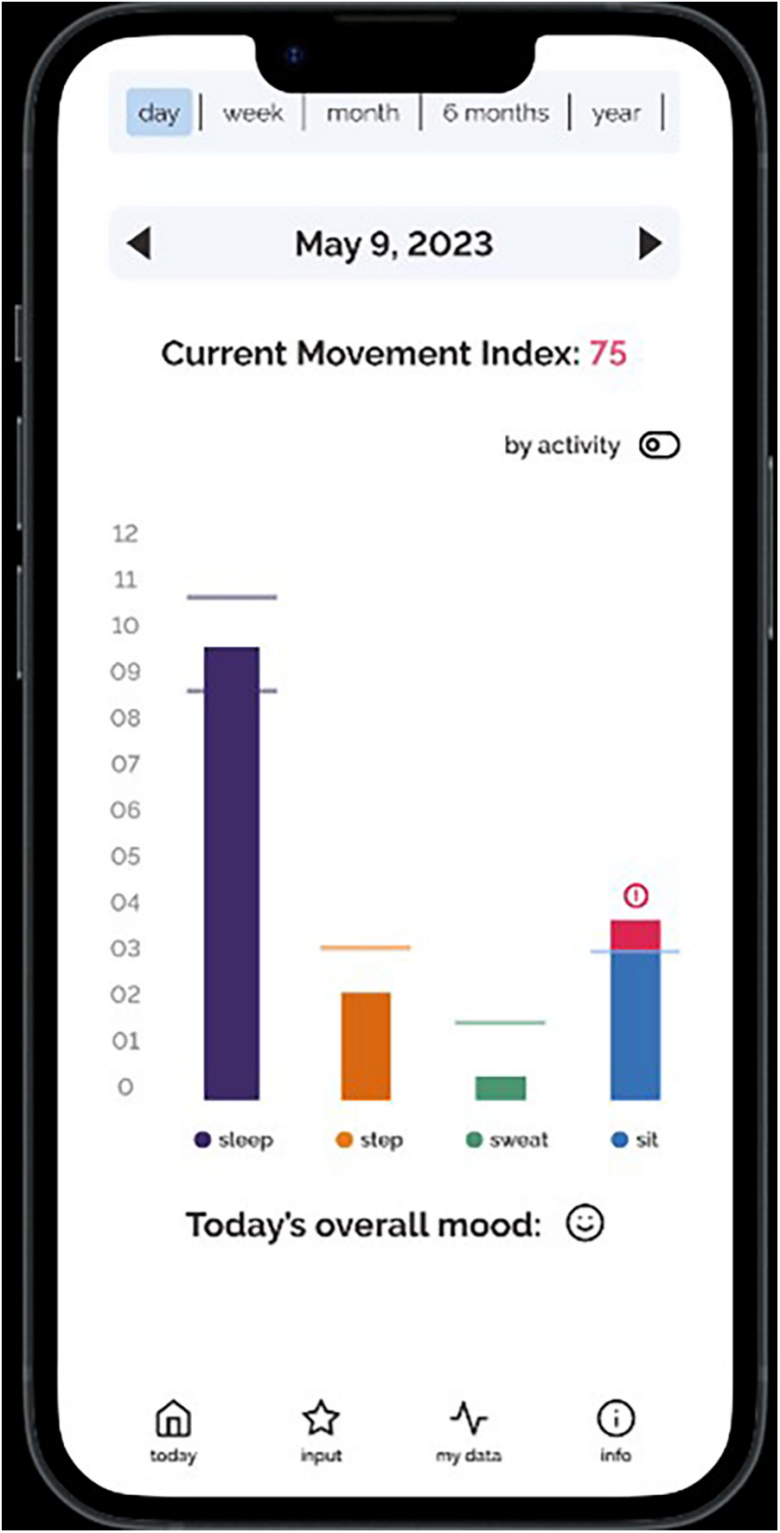
Screenshots of the Movement Index prototype.

**Figure 2:**
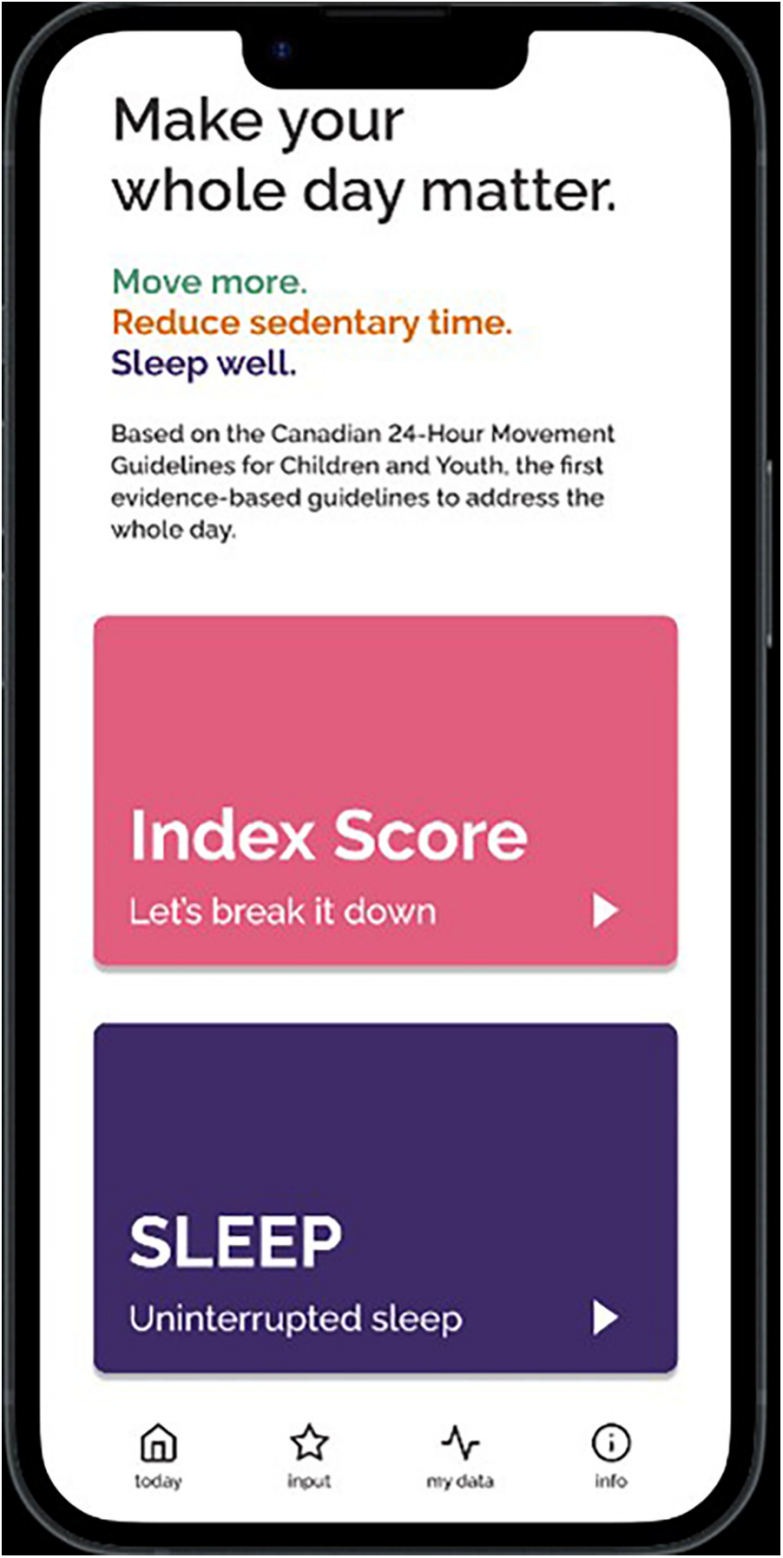
Screenshots of the Movement Index prototype.

**Figure 3:**
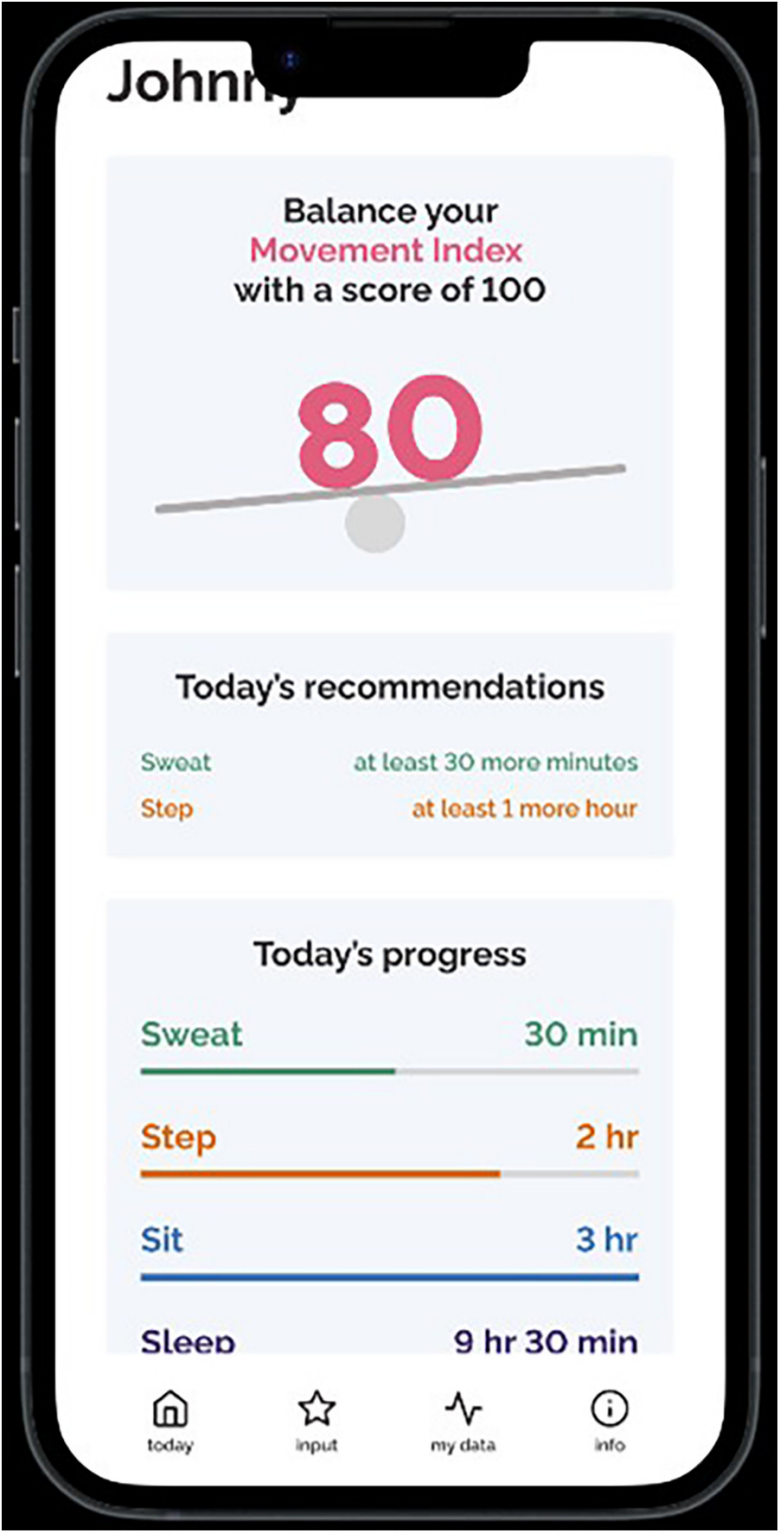
Screenshots of the Movement Index prototype (continued).

**Figure 4:**
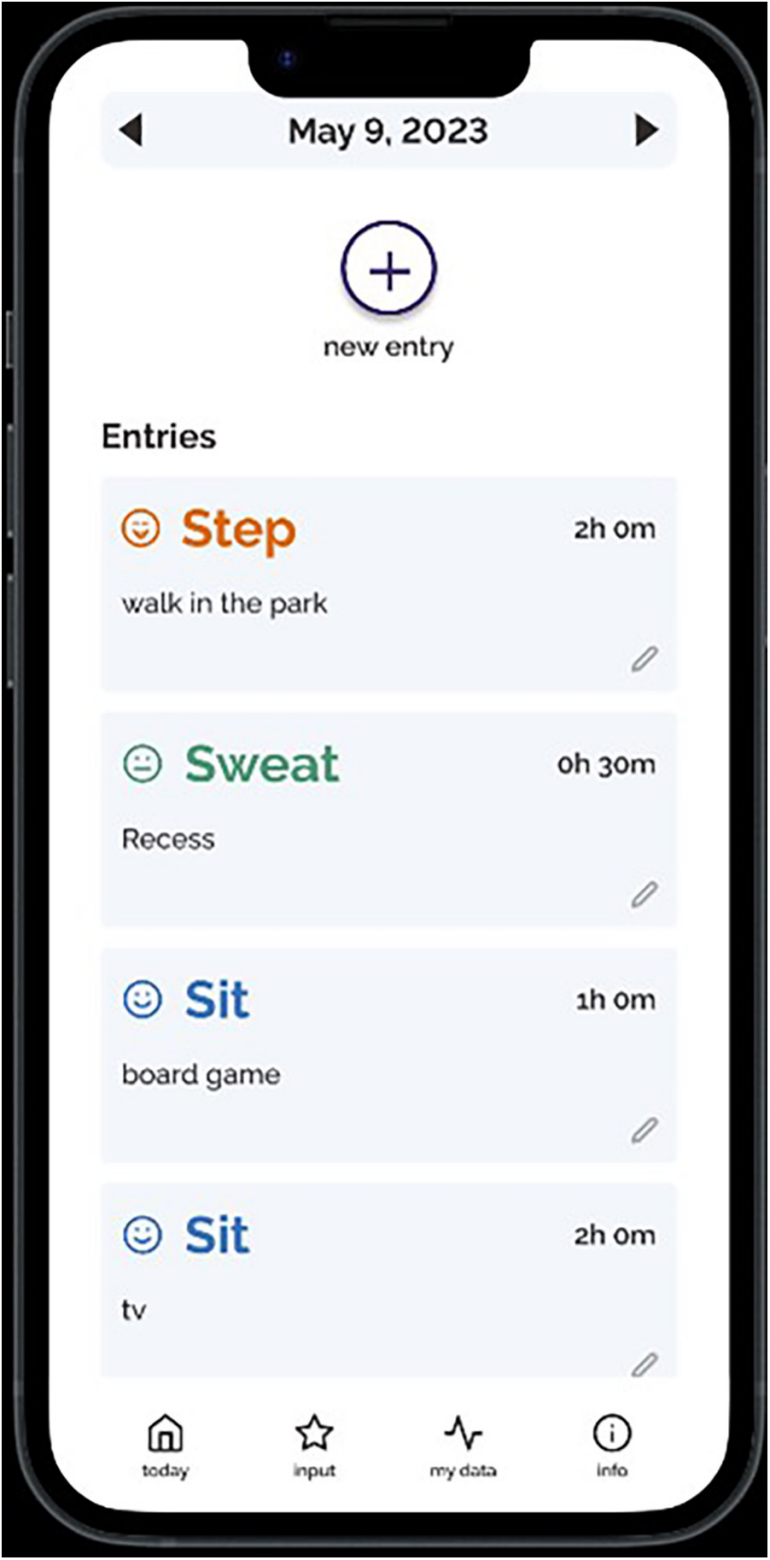
Screenshots of the Movement Index prototype (continued).

Individual semi-structured interviews were conducted over Zoom. The interviews were on average 41 min and 24 s in duration and were recorded and transcribed verbatim. The interview guide was developed based on all seven TFA constructs that explored participants’ perceptions of the Movement Index (see Supplementary File). The interview guide was first pilot tested with a mother, and the results were used to refine the questions to further target the different TFA constructs and placed more emphasis on a holistic exploration of the concept of the Movement Index. The open-ended questions allowed participants to expand on their answers, leading to an in-depth exploration of each participant's experiences and perspectives. The lead author navigated and walked through a prototype of the Movement Index (see Figures 1–4) on an interactive website to help guide the interviews. Participants were compensated with a $25 Amazon e-gift card after the completion of the interviews for their time and contribution to research and this aligns with standard practice at the authors’ institution.

### 2.3 Data analysis

A deductive thematic analysis was conducted using the TFA as a guide, while maintaining theoretical flexibility for inductive analysis. The general structure of the six-step process outlined by Braun and Clarke (29) was followed. First, the lead author familiarized himself with the data during the process of data collection and transcription. The lead author re-read the dataset to gain a deeper familiarity with the data and identified key information that may or may not fit into the seven constructs of the TFA. Next, initial codes were generated. Deductive coding was first used with predetermined codes from the TFA, and information was found from the data that would fit into the range of the codes. Inductive coding was also used to take an alternate approach and develop codes that did not fit any of the TFA constructs. The lead author actively sought out both semantic and latent codes, which covered superficial content conveyed by participants and deeper hidden meanings of data and assumptions (30). After all the relevant data were coded, a constant comparative method (31) was used to analyze and compare codes with each other to look for similarities and differences. Similar codes were categorized under the same subtheme and eventually under the same theme (constructs of the TFA). While the analysis started with the TFA framework, the constant comparative method allowed flexibility in adapting and refining the framework based on the data. Next, the themes were reviewed to ensure they were meaningful and could address the research questions. Themes were revised, and those that were irrelevant to the research questions were removed.

### 2.4 Trustworthiness

To enhance trustworthiness of the study, the lead author engaged co-authors as “critical friends” throughout the analytical process who collectively and collaboratively offered valuable insights and fostered meaningful discussions on the data collected (32). The lead author remained reflexive and reflected on how his personal experiences and values may impact the results. A reflexive journal was used throughout the study to keep track of thoughts, feelings, and decisions. Last, to ensure the transferability of the study to other contexts, a detailed description of the participants' responses and interpretation was provided to facilitate transferability (33).

## 3 Results

First, participants' perceptions of the 24HMG were explored. Then, themes were developed from the seven constructs of the TFA, including perceived effectiveness, intervention coherence, affective attitude, burden, opportunity costs, self-efficacy, and ethicality. Participants' names were removed and replaced with pseudonyms (M: mother; F: father) to ensure privacy and confidentiality. Last, feedback and recommendations are provided for the future refinement of the Movement Index (see Table 3).

**Table 3:** Acceptability of TFA constructs and recommendations for refinement of the Movement Index.

TFA Construct	Acceptability	Recommendation
Perceived effectiveness	✓ ✓	Highlight role of the Movement Index as a collaborative “conversation starter” between parent and child about what a healthy 24 h looks like.
Intervention coherence	✓ ✓	Provide user-friendly information on how the Movement Index score is calculated and how it is tailored to the individual child.
Affective attitude	✓	Include supportive feedback for parents to mitigate stress and avoid feelings of failure. Reinforce behaviour change as not being ‘all or nothing’ and that balancing behaviours is the goal rather than meeting individual behaviour benchmarks *per se*.
Ethicality	✓	Explore other modes of delivery without the use of activity trackers such as web-based platforms based on manual data entry and children's recall.
Burden and opportunity cost	?	Develop the Movement Index's short-term functions (e.g., short term monitoring and educational tool). Provide information regarding length of time commitment.
Self-efficacy	?	Incorporate social features and incentives to enhance engagement. Take advantage of initial novelty feeling and high motivation among users, and help users understand balance of movement behaviours during first two weeks. Add motivational features such as tailored reminders, graded tasks, and progress milestones.

Two checkmarks represent acceptable, one checkmark represents mostly acceptable, and question mark represents mixed acceptability.

### 3.1 Perceptions of 24HMG

In general, parents were familiar with the overall concept of guidelines for movement behaviours, such as physical activity and sleep guidelines. They believed that their children need to be active, get enough sleep, and reduce daily sitting. However, regarding specific awareness of the 24HMG, only two parents could recall them. Both parents engaged with the 24HMG in their work setting. For instance, one participant is a grade nine physical education teacher that uses the 24HMG as part of her classes, and she mentioned: “*I have seen this…so actually, I shared these exact graphs that you're showing with the four categories… with my grade nine Phys. Ed. students.” (M3)*

Another participant works as a dietitian, and her responsibilities include using the 24HMG to promote healthier lifestyles and support people with behaviour change. She mentioned: “*I use them, and I refer to them often for my work. Again, not specifically for the kids, I haven't looked at those for a while.” (M2)*

While other parents knew that there were movement behaviour guidelines and could recall some variations of the guidelines, they could not recall seeing the specific 24HMG. For instance, F10 mentioned how he had never seen the guidelines in this format that made distinctions between physical activity, sedentary behaviour and sleep. Another participant mentioned similar thoughts:

I have probably heard by a different name, I can't remember the exact name, probably just like child activity or activity guidelines. They were more focused on how much sweat type of activity they should engage, like active movement engagement that is required on a daily or a weekly basis. So it's very similar. (F6)

None of the participants were able to recall the specific physical activity, sedentary behaviour, and sleep recommendations of the 24HMG. Even the two parents aware of the 24HMG could not articulate the recommendations.

After explaining the specific components of the guidelines to the participants, all the participants agreed with the importance of having such guidelines and their potential contribution to their children's healthy lifestyles. For instance, one participant mentioned: “*So it’s quite important for us, for my wife and I, to ensure that our kids are meeting those kinds of guidelines.” (F7).* Another participant mentioned how the 24HMG is important to her as a parent as it allows her to make connections between meeting guidelines and specific behaviours of her child:

Being able to see the connection of how balanced all those things are, to how my child is doing during the day kind of thing, both in school or at home; are they fighting more with their siblings or irritable or are they really focused? I feel like that's really beneficial for parents, like if their kid is so unbalanced with those things, getting them balanced could be the answer to a lot of things. (M6)

While few of the parents were aware of the 24HMG, most expressed a strong interest in learning more about them and perceived them to be important and useful. Overall, this low awareness perhaps underscores the need for sustained educational efforts and additional knowledge translation tools, such as the proposed Movement Index, to disseminate the 24HMG to parents.

### 3.2 Theoretical framework of acceptability

The initial reaction was generally positive when the Movement Index was explained, and the prototype was shown to the participants. Participants found the concept of the Movement Index to be enlightening, intuitive, and potentially helpful to them as parents. The findings are categorized into the seven constructs of the TFA.

#### 3.2.1 Perceived effectiveness

Perceived effectiveness refers to the extent to which an intervention is likely to achieve its purpose. Overall, participants perceived the Movement Index as potentially effective for the following reasons: acting as a knowledge translation tool for parents through increasing education on movement behaviours, instilling accountability, and enhancing opportunities for teamwork with their children.

##### 3.2.1.1 Increasing awareness and knowledge

Participants strongly believed that the Movement Index would be an effective knowledge translation tool that educates them and raises awareness regarding the 24HMG. Because their initial awareness of the 24HMG was mostly non-existent, the Movement Index could play an important role in changing that, consequently helping them to take action to support their children's movement behaviours:

It is very visible, what the goal is each day in each category specific to their age. That's really great because it makes you aware of what you're aiming for, right? It's hard to make your kids do something if you don't even know. (M4)

Greater visibility of movement behavior recommendations may prompt parents to reflect on daily routines and take more intentional actions to support their children in meeting the 24HMG. Another participant saw the Movement Index as a tool that bridges the gap between the guidelines (research) and practice:

I definitely think that puts that information into practice and makes good use of that information because it's nice to know it, but if no one uses it, what's the use of all that research kind of thing? So yeah, I do think it is a good bridge to do that. (M6)

The Movement Index could also have a role in increasing parents' knowledge about their own children's movement behaviours each day, as F2 mentioned: “*It’s helpful that this app provides immediate feedback as to where they're at for the day, without actually having to do any digging through stats or anything.”* In doing so it would also allow ongoing monitoring: “*I think the “my data” and the “historical values” are pretty helpful. You can kind of see how you're progressing over time.” (F1)*

The following participant mentioned a similar notion about increased awareness of their children's sedentary behaviours, and this awareness could motivate them to try and change their children's behaviours:

It will be very helpful for parents to see how much their children actually sit, and I think they will be driven to change that and drive them to get their kids to shift towards more of step or sweat. And plus, I think parents have so much say in how much their kids are sitting as opposed to other activities, so I think it'd be really helpful with reducing that sedentary time. (M3)

It was apparent that a significant perceived benefit of the Movement Index was acting as a potential knowledge translation tool for the 24HMG. This would educate parents, increase their knowledge of the guideline recommendations, and bring more awareness to their children's movement behaviours and patterns.

##### 3.2.1.2 Accountability

Several participants mentioned accountability as a perceived benefit, believing that this app could help them be more accountable for their children's movement behaviours. As one participant described:

This app would help with accountability both as a parent and as a child towards a commitment to being more physically active, and therefore reducing things like diabetes and cardiovascular disease down the road. I think that as soon as parents see this, and start using it…not only is it going to hold the kids accountable, but it's going to help hold them accountable. And, you know, most parents want their kids to be healthy. (F2)

Another participant mentioned how this app could act as a reminder for them to act on their children's movement behaviours, constantly prompting them to try and help their children achieve a healthier balance of movement behaviours: “*It keeps confirming or reaffirming every day that you need to get them out. They need to get out and do different types of activities. Or, at the very least, get them moving.” (M10)* Similarly*,* M5 described how she might feel more compelled to take her son out for an activity at the end of the day if she noticed the hypothetical Movement Index was low.

There was consensus that in using the Movement Index, parents might become more accountable for their children's movement behaviours, thus helping them progress towards the 24HMG and achieve the optimal balance between movement behaviours.

##### 3.2.1.3 A conversation starter

Participants described the Movement Index as a conversation starter with their children about their movement behaviours and what changes might be beneficial:

At the end of the day, like just before my son goes to bed, for example, we could sit down and go okay, well today, what did you do? And it gives us sort of a focused approach to discuss, today we did this, and we did this, and we did this. So did we do enough? Or do we need to do more? It provides a discussion point for him and I at the end of the day. (F2)

M5 offered a similar opinion on the app in that it may provide her with a starting point of conversation to review what the child did well during the day, and where changes might be helpful:

At the end of the day I can be like, whoa, what happened here today? Why were you sitting for six hours at school? Oh, well, indoor recess, blah, blah, blah. Like, you know, it's a conversation point for me too. And then I can be like, we're gonna go for a bike ride tonight as a result or something. I think that's valuable for sure. (M5)

Another participant mentioned the importance of having their children's involvement in using the app, and that it needs to be a collaborative effort in shared goal setting to bring change in behaviours:

I think it's important that somehow there's a way for them to be into it, too, then it's gonna help the parents stay motivated. Especially if it's work on my end, I want my kids to get value out of it to adjust whatever needs to be fixed. (M8)

Overall, using the Movement Index collaboratively with one's child was considered an opportunity for both parents and children to become more aware of their movement behaviours and potentially plan how to modify them to find the optimal balance.

#### 3.2.2 Intervention coherence

Intervention coherence refers to the extent to which the participants understand the Movement Index and how it works. Since the Movement Index is still a prototype, there are still many uncertainties including personalization and Index score calculation. However, most participants perceived the concept of the Movement Index as logical and clear, and reported having a good understanding of its purpose. For instance, this participant showed her understanding of the functions of the Movement Index:

I like how you're looking for a balance of the four categories, not just saying going for one thing at a time and saying, hey, you didn't sweat enough today or something. So I think that’s really cool, and innovative to find that balance of all four things. (M1)

The following participant also showed her understanding of how the Movement Index is trying to demonstrate the balance of movement behaviours and how the “teeter-totter” visual is used to depict this:

I understand that you sort of want to balance the active stuff with the inactive stuff. And that is not to say that, that sleep and sitting aren't important. But they need to be sort of moderated and, as you say, balanced against the active stuff. The horizontal thing makes a lot of sense since it is trying to be balanced. (M3)

There were a few instances where participants' articulation of the Movement Index score was less clear and more focused on meeting the individual guidelines. For example, the following participant illustrated this misconception:

Yeah, it does make sense, for your example I see that the step guidelines are not being met, and the sit guidelines are being exceeded which contributes to not being as high of a score, but I guess if all of these guidelines are met then the score would be close to 100. (M4)

Participants also brought up several areas of uncertainty. One participant was uncertain about making the app more individualized since each person will have different circumstances:

That's where I'd be like, that's a bit of a challenge. To me, depending on a certain child's starting point, it could be different, right? For example, you have a child who is morbidly obese to begin with and has a lot of health issues. For them, you know, 20 min of sweat in a day might be ideal to begin with, because that's all they can physically tolerate. For them, it might be demotivating to see their balance score so low when they're literally trying their hardest. Whereas, for example, if you take someone like my son, who has ADHD, and he literally can't stay still, and he needs a good two, three hours of sweat. So I don't know how customization would be like in that sense. (F7)

A minority of participants also questioned the app's technicalities, particularly the syncing of movement behaviours. One participant questioned whether it is practical to be able to synch all the movement behaviours into the app and questioned the accuracy of the data if it did get synched into the app:

My question would be around kind of how do the devices sync data? And how challenging is it to kind of get it all set up? Since there are so many things to keep track of during the day, like sleep, physical activity, and sitting, is it possible to get all the information accurately? (F8)

The participants had an overall coherent understanding of the Movement Index and its functions, with some general areas of uncertainty around the syncing of data, Index score calculation, and the individualization component. At the current stage of the prototype, these are still areas of uncertainty and are being developed by researchers.

#### 3.2.3 Affective attitude

The construct affective attitude describes how the individual feels about using the Movement Index. Feelings and emotions towards using the Movement Index include a sense of curiosity and appreciation for its simplicity but also a potential source of stress.

##### 3.2.3.1 Sense of curiosity

Many participants were curious about the Movement Index, their children's movement behaviours, and how using the app could positively contribute to their children's health. The following participant mentioned she would be curious to try the app out to see if there is any difference between perception (e.g., that their child is “physically active”) and reality:

I'm curious if there's a discrepancy between what the current situation is and what those recommendations are? We tend to overestimate in our minds, that we do certain things, and we underestimate other things. But is that really the case? (M2)

Another parent echoed this sentiment and emphasized knowing more about the children's sedentary time through the Movement Index:

“I'm curious to see, because I have an idea of my children’s behaviours, but sometimes you don't realize how much sedentary time they might be having. So I thought it would be interesting to know through this.” (M8)

M6 cited her child's academics as a source of curiosity in using the Movement Index; she would want to see whether her child's academic challenges could be attributed to movement behaviours:

I'm just thinking about my one son struggling to focus on his worksheets in school. It would be kind of neat to put his information in this and be like, okay, is his physical activity lacking? Could this be part of the problem? Why he's having trouble doing these worksheets in the winter? (M6)

M5 even said that using this app could act as a “*wake-up call”* since she believes that through using this app, she could learn a lot more about her children's movement behaviours than what she currently knows. Another participant mentioned how, despite believing it is better to monitor her child's health proactively, she thinks she will use it as a reactive tool to explore the movement behaviour profile of her child after something has happened:

I guess I would probably use it after the fact that my kid has a problem. Like it's better if you use it proactively, I guess. But for me, I think after a problem occurs, now I'm going to use it for a few months out of curiosity. (M6)

Many participants described a sense of curiosity when discussing the Movement Index, highlighting the app's potential role in providing short-term, informative feedback on their child's movement behaviour.

##### 3.2.3.2 Simplicity

Participants perceived the concept of the Movement Index to be simple and easy to understand, as several remarked: “*It seems like a simple and straightforward app that I could use easily” (M8)* and “*The concept seems pretty intuitive and doesn’t look complicated, so that's good to use.” (M2)* The concept of having one single summarizing Index score was also praised by participants, as it provided a simple, useful summary of each day's combination of movement behaviours: “*You know, it looks good. It gives you a snapshot in an instant and lets you know that there's a target number and that you haven't quite reached the target yet.” (F5)* Another participant echoed the previous point: “*I love how the score combines all aspects of the guidelines, and when you hop on the app, boom, there it is, and this gives you lots of information in just one simple number.” (M8)*

The Movement Index's perceived simplicity is likely to benefit potential future uptake by parents. It may also increase their self-efficacy in using the app for its intended purposes (see section 3.2.6).

##### 3.2.3.3 A source of stress

There were three participants who expressed concerns that using the app might induce negative emotions such as stress and guilt in certain instances. Parents might become overly stressed if their children do not meet the recommended guidelines, which could potentially induce guilt that they were not a “good parent”. For example, the following parents stated: “*Parents are going to look at this and go, okay, I'm going to do this now. I just find it almost guilt-inducing” (F2) and “You don't want somebody so stressed out, and their stress goes through the roof in order to meet the threshold or close the rings or whatever it is that they're trying to do.” (M2)*

Similarly, one participant mentioned that if he consistently saw low scores on the app, he might think he has failed as a parent to keep his children healthy, which could add extra stress and burden to himself as a parent.

And for me, if I saw a score of 20, and it was like this really, really uneven thing, I feel like I'm failing as a parent. I would be like I'm a horrible parent, I'm failing my child, and I would feel so bad inside. (F3)

One factor that contributes to this stress could be the difficulty in meeting the guidelines, especially in the sedentary behaviour category. This was exemplified when parents described the sedentary guidelines as “*unrealistic” (F1)* and “*most challenging” (M8).* Another participant explained this challenge:

As for the last part, the sedentary behaviour, no more than two hours of screen time. That's a little bit hard to achieve right now. Because as a busy mom, sometimes I'm not able to give him that much attention, one-on-one attention. So when I'm busy, I usually give him the iPad. And that's usually three, four hours a day. (M4)

While caution is required in considering such inferences, an important concern about tools like the Movement Index is warranted for their potential to be a source of stress for some parents.

#### 3.2.4 Burden

The burden construct refers to the perceived effort required to participate in a particular intervention. Generally, participants acknowledged that using the Movement Index might not be much of a burden. However, the nature of the data entry required was frequently described as a potential source of burden.

##### 3.2.4.1 Data entry

Two potential data entry options were explained to the participants: manual and automatic. The manual data entry option was seen to have a high burden among participants, as they perceived this option to take a lot of effort, which would decrease their motivation to use the app: “*If you had to actually write out every input, unlike the pre-generated, it would be tough. I don't think I would be incentivized to type out every single thing.” (F3)*

The burden of data entry was perceived as higher for parents who worked long hours, where there were other priorities for the parents before tracking their children's movements. Moreover, if participants had multiple children, it would become increasingly challenging and impractical to enter the data for all their children manually: “*With four kids, there's no way I'm following all four of them around all the time to be able to give you accurate numbers.” (F1)* Another participant shared a similar opinion:

I feel like inputting everything might be kind of tricky. I guess if it's just for one kid, and it's like, try and get to the root of a problem, it would take effort, but maybe doable. I think if you're trying to do multiple kids at the same time…it could be trickier with that. (M6)

When the automatic data syncing option was mentioned, participants were much more in favour of having this option available as it was perceived to be less burdensome. For instance, M7 mentioned: “*It's a good tool if you're really concerned. But otherwise, I think I wouldn't bother. But if I could upload it automatically, like use data from a watch or something then, absolutely I would use it.”*

Some of the parents suggested that perhaps a combination of both data entry methods would be the most effective and provide the best balance between data entry and burden:

For me, I would say sync data with the device. But having the other one is a good option too. Because I realize that not everyone is in a position to afford a fitness tracker and stuff. And then there's remembering to remind the kid to wear it. So it's always good to have a manual backup. (F9)

There was consensus that the manual data entry option was too burdensome, which could negatively impact the acceptability of the Movement Index. Automatic data entry was highly favoured among participants, suggesting a need for some kind of data syncing with the app or at least a combination of manual and automatic data entry methods to minimize the burden.

#### 3.2.5 Opportunity cost

Opportunity cost refers to the extent to which benefits, profits, or values must be sacrificed to use the Movement Index. This construct is related to the previous construct of burden as the type of data entry was associated with different opportunity costs. Concerns about financial costs were also raised, and this construct reflects these concerns.

##### 3.2.5.1 Financial cost

Due to the burden of the manual data entry method, the need became apparent to parents for automatic data syncing to inform the Movement Index. To make this possible, families would need to own a wearable “fitness tracker” to monitor children's movement behaviours. This could be an opportunity cost for lower socioeconomic families in which the “*families have to give up some money that they need for other aspects of their lives in order to participate in this intervention, which makes it harder for them to participate.*” *(M9)* The following participant expressed concerns over the financial cost of a wearable device and how it would limit certain families' participation:

I think that would be an impediment, Fitbits aren't cheap and giving one to a six-year-old is not advisable, my six-year-old loses everything. But I think that's going to be a challenge for your study is finding families because the families who can afford to provide their five to eleven-year-olds with Fitbit [a wearable fitness tracker], or Apple watches or something electronic and able to track movement, sleep quality, and so on, who also have kids who are willing to wear a watch while they sleep? Like, yeah, your data is primarily going to be self-reported probably. (M9)

Irrespective of socioeconomic class, cost might still pose an issue if there is not strong motivation. Parents might not want to go out of their way to buy an activity tracker so that they can use the Movement Index. M2 mentioned: *“The feasibility of me spending money to put an activity tracker on my kid's wrist, I don't know if that would be high up on our priority list.”*

##### 3.2.5.2 Time/effort cost

Consistent with the burden construct, the major opportunity cost in using the Movement Index would be the time and effort needed for monitoring their child to make informed decisions on the data to provide in manual entry. Comments such as “*people are very protective of their time,” (M5) “parents are so busy with their work and family, and their time is very valuable,” (F3) and “you have to be careful about what you're adding on to people's work currently” (M2)* demonstrate how important and precious time is for people, especially for parents. Thus, time and effort costs would become a major obstacle to making a habit of using the Movement Index:

As parents, we definitely want the best for our children, but one downside would be finding the time to make it a habit. It can be so chaotic from the second you wake up to the second, you know, everyone's tucked into bed. So I think the time commitment would be the biggest kind of obstacle for parents. (M3)

Therefore, parents might feel they need to allocate time to use the app that would otherwise be spent on other productive activities, such as household responsibilities or direct interaction with their children. In contrast, some parents strongly believed that the time and effort to use the Movement Index would be worth it for the benefit of their children. This was acknowledged by two parents: “*I don’t see how parents couldn’t find a few minutes of their lives for the overall benefit of their children's health” (M9)* and *“I think it's doable, I’m sure I can find some time to do after work to help my children be healthier.” (F4)*

While parents were supportive of the Movement Index, the time and effort cost must be considered. It is important to note that perceptions of the time/effort cost of using the Movement Index depended largely on whether data entry would be manual or automatic.

#### 3.2.6 Self-efficacy

Self-efficacy refers to participants' confidence in using the Movement Index effectively. In this case, self-efficacy was reflected in terms of self-efficacy in-app navigation, manual data entry, and long-term engagement.

##### 3.2.6.1 Self-efficacy in app navigation

Most participants expressed high self-efficacy in their ability to navigate through the Movement Index (as reflected in the mock prototype) and to use it for its intended purpose. For instance, the following two participants demonstrated their self-efficacy in app navigation: “*It looks easy, like I could navigate it. My kids could probably navigate it on their own. Yeah the concept seems good and user-friendly” (M7)* and “*I use apps for everything, so I don't see this one being complicated… I think the user interface is pretty simple.” (M9)*

Like the sub-theme of simplicity within the affective attitude construct (see section 3.2.3.2), participants generally described the Movement Index as intuitive, easy to understand, and user-friendly, contributing to high perceived self-efficacy.

##### 3.2.6.2 Self-efficacy in manual data entry

Despite the participants' high self-efficacy in the navigation of the app, their self-efficacy in the manual data entry and knowing what their children did during the day to enter data was relatively low. The following participant believed it would be very challenging for manual data entry when she is not with her children, particularly in the sweat, step, and sit categories:

It's a challenge because I'm not with my kids, especially during weekdays, for most of the day. So I don't know what that would look like, as far as like, what would be involved with monitoring. Sleep is definitely something that I don't see any challenges. But the other three aspects, especially sedentary behaviour, if I'm not physically with them, how do you go about actually monitoring it? (M2)

The following participant also shared his view on manual data entry which reflected his low self-efficacy:

But I'll be honest, it might be a difficult expectation to make if it requires the manual input of data on a regular basis, on a consistent basis. I don't think I'm able to do that, so some type of data feeding is needed to make it work. (F6)

##### 3.2.6.3 Self-efficacy in long term engagement

Participants displayed low self-efficacy in the long-term engagement with the app. This was demonstrated from F3's comment: “*It’s a great idea to learn, I doubt that I will be engaged with it very long, other than maybe taking a look at it, and never opening it again.”* The challenges of using the Movement Index daily were also brought up:

“I think opening the app is the biggest challenge. Because when you're looking at trying to change people’s behaviours, if they're not in the mode of it, or there’s like, no huge motivation for them to do it, then it’s harder for them actually to want to open the app.” (F4)

No information was provided to the participants about how often a user may need to engage with the Movement Index app. In exploring self-efficacy, it became clear that this will be an important consideration in its future development.

#### 3.2.7 Ethicality

Ethicality is the extent to which the intervention fits with an individual's value system. Ethicality was considered in terms of alignment with healthy living values and equity implications.

##### 3.2.7.1 Healthy living values

There was a consensus among the parents that the Movement Index aligned with their values of healthy living. They want their children to be healthy, and if this app helps to achieve that, then it would be in line with those values:

The purpose of collecting this data is to improve the overall healthy balance of active lifestyle of youth rate of these age groups. Just seeing the benefits and the objective of the purpose of this app, I think it creates a lot of really huge positive contributions to the youth group, which I value. (F6)

Moreover, one participant mentioned how the Movement Index could help to instill awareness and a sense of responsibility among parents, which aligns with their values of “*wanting their kids to be healthy and happy.” (F2)* This point relates to the accountability sub-theme under the perceived effectiveness construct; the Movement Index might hold participants accountable to their values of healthy living that include being active, reducing sedentary time, and getting sufficient sleep.

##### 3.2.7.2 Equity implications

Some participants brought up the topic of equity and exclusion. For instance, one participant mentioned that not every family can afford a tracking device, which is crucial for data entry: “*I guess the only things that kind of pop in mind is like equity. not everybody in your family can get an Apple watch or digital device, right?” (F3)*

The privilege issue was highlighted as the Movement Index was perceived by one participant to be tailored towards more privileged families. They can afford electronic tracking devices, but most likely can also afford other aspects of a healthy lifestyle, such as access to gyms or being able to enroll their children in organized sport and recreation:

The Fitbit idea is great. But it will be a financial impediment. And I mean, then you have to look the families that can afford to have a Fitbit. What privilege do they have? If they can afford that, can they afford a gym membership? Or can they afford to put their kids in clubs, where other families who can't afford a Fitbit won't be able to afford those things? You're going to have a divide there, like you're not going to get a general population, you're going to get a socio-economic level that supports the strata that can afford Fitbit, and all the privilege that comes with that. (M9)

Given assumptions made by some participants that lower-income families are less likely to be able to use the Movement Index, the following participant reinforced the idea that the Movement Index might exclude such families:

If you put a health or social determinants of health lens on things, the people that need the most help, is this the best way to help them? And I don't know because, again, you know, are those families going to have the technology and everything else to be able to use something? You're probably not necessarily going to get the lower socioeconomic status. (M2)

There is clear potential for the Movement Index as conveyed in the interviews to be inequitably effective if costly syncable trackers were also required. This holds significant implications for the next steps of its development.

## 4 Discussion

This study examined the acceptability of a proposed Movement Index, an app-based 24HMG knowledge translation tool. While parents acknowledged the importance of the 24HMG, they had low awareness and knowledge about those guidelines. This highlights the need for increased active dissemination efforts of the 24HMG and the development of practical knowledge translation tools like the Movement Index to increase awareness of the guidelines while also helping parents support their children in balancing their movement behaviours. In examining the acceptability of the Movement Index, there is evidence that it is acceptable, particularly in terms of its perceived effectiveness as a knowledge translation tool, simplicity and coherence, and alignment with healthy living values. However, further consideration will be required regarding the burden and opportunity cost of manual data entry and some ethical considerations, including surveillance and equity implications.

Current findings about low awareness of the 24HMG were unsurprising. Simply producing guidelines is insufficient for generating awareness among the public. More active dissemination efforts are needed if one assumes that awareness and understanding are precursors to behaviour change among parents (e.g., providing encouragement) and children (e.g., reducing screen time). The Movement Index has been proposed as a novel tool for promoting this awareness while also helping parents and children understand the importance of the inter-relationship between all movement behaviours throughout a healthy day. With a prototype of the Movement Index developed as a prompt for the interviews, this qualitative study performed an in-depth evaluation of the acceptability of the Movement Index using the TFA. While the TFA has been previously used in studies examining the acceptance of mobile health apps [e.g., Chen et al. (34) examined the acceptance of everyday use of health apps in adolescents], this is the first evaluation that used the TFA to assess a physical activity and movement behaviour app.

### 4.1 Analysis of TFA constructs

An evaluation of the seven constructs of the TFA suggests that the Movement Index was acceptable on two constructs (perceived effectiveness, intervention coherence), mostly acceptable on two constructs (affective attitude, ethicality), and had mixed acceptability for the three remaining constructs (burden, opportunity cost, self-efficacy). Recommendations for the future development of the Movement Index are identified (see Table 3 for a summary).

The effectiveness of the Movement Index as a knowledge translation tool was perceived to be high, as it can raise awareness of the 24HMG and close the gap between research (24HMG) and practice (monitoring). Becoming more aware of the 24HMG is foundational to parents effectively taking action, such as through positive role modelling to support their children's balance of movement behaviours (35). The Movement Index could potentially make guideline information more personalized, practical, and accessible to change behaviours at the individual level. Knowledge translation tools tailored to the audience's specific needs and context are more effective (36). The Movement Index emphasizes this personalization, as each individual may require a different balance of movement behaviours to achieve optimal health outcomes.

Furthermore, the Movement Index was perceived to be effective in holding parents accountable for their children's movement behaviours and serving as a conversation starter, which can lead to a collaborative effort to achieve a healthier 24 h. Previous research has emphasized the importance of having a collaborative approach between children, parents, and other partners, such as educators and doctors, in meeting the 24HMG (24). This collaborative approach can help children develop an awareness of healthy movement behaviours and provide a foundation for lifelong healthy movement patterns (37).

Intervention coherence was another positively perceived construct, as most participants understood the intent of the Movement Index to promote a healthy balance of movement behaviours. Some app-specific uncertainties did arise, including how the Movement Index score is calculated from the background data and how it would be tailored to different individuals. This is currently not finalized at this stage, as researchers are currently collecting background data towards “precision 24-h movement behaviour recommendations” to make personalization and score calculation possible (38). Findings regarding this construct suggest that it will be important to provide information on how the Movement Index is calculated and how it will be tailored to each individual child.

Overall, participants had positive affective attitudes toward the Movement Index, appreciated its simplicity, and were curious to try it out. However, some participants suggested that the Movement Index could be a potential source of stress. This was similar to previous findings regarding movement behaviour guidelines becoming a source of stress for parents, inducing feelings of guilt that they are not doing a good job parenting if they cannot help their children meet them (24, 39). The Movement Index should reinforce notions that behaviour change is not “all or nothing”, and that balancing behaviours is the goal rather than meeting individual guidelines *per se*. This might alleviate the potential of the Movement Index being a source of stress and guilt for some parents.

The burden and opportunity cost received mixed perceptions due to the perceived time and effort required for manual data entry. Considering the context of busy family and work schedules, this burden itself might be another source of stress. Parents, particularly those with fewer resources, experience heightened stress compared to non-parents (40). Thus, using the Movement Index could be seen as “one more thing” to worry about, although this perception heavily depended on the type of data entry and the duration of the engagement. Moving forward, consideration should be given to reducing the perceived burden and opportunity cost of using the Movement Index. If manual entry is required, it will be necessary to develop functionality such that the tool could be used as a short-term educational and monitoring tool. The Movement Index is still at a conceptual stage, and how, or how often, the app may need to be used has not been considered. Information regarding the length of time commitment to using the Movement Index will need to be clearly provided to users in the future.

Participants showed high self-efficacy for navigation through the Movement Index, which could be attributed to the simple and user-friendly features of the prototype. However, participants did not perceive high self-efficacy in the manual data entry and with long-term engagement. This should be interpreted cautiously, as the Movement Index does not exist yet, and the participants' view on their self-efficacy in its use is rather speculative. Maintaining engagement is a common problem with mobile health apps and wearables (41–43), and suggestions to increase user engagement include incorporating personalization, social features, and incentives (44). Furthermore, motivation is usually high during the first few weeks of use, and this “window of opportunity” could be capitalized to try and integrate the use of wearables into the user's daily life (45). The Movement Index could take advantage of this “window of opportunity” and be designed in a way that two weeks of usage would provide an educational purpose for understanding the balance of movement behaviours, and pinpoint changes that should be initially focused on (e.g., reallocating time from sedentary behaviour to light physical activity).

Regarding the ethicality of using the Movement Index, it was perceived to align well with the participants' active and healthy living value systems. This is an important aspect of the Movement Index, as mobile health apps that reflect users’ personal value systems are more likely to support long-term usage and lead to better health outcomes (46). However, some participants raised equity concerns, such as the potential for a technological divide for sub-populations who may not have the financial resources to afford technological tools (47). This could prevent all families from benefiting from the Movement Index even though some groups (e.g., families of lower socioeconomic status) may benefit the most. An important implication is that the Movement Index may need to be tailored to be accessible to families of lower socioeconomic status. For instance, different types of delivery could be considered, such as web-based platforms, which allow for manual data entry to explore the balance of movement behaviours based on children's recall. An example is demonstrated by a University of South Australia study featuring an interactive web application called “Your Best Day” (48). Through manual data entry using time reallocation sliders, this web application allows users to explore how reallocating time among daily activities can impact different health measures such as fitness, mood, and cognition.

While participants from the current study had low concerns about their children using tracking devices, their use by children still warrants exploration from both a theoretical and ethical standpoint. A study of one hundred teenagers in the United Kingdom on the use of Fitbits found resistance from the youth as they believed this kind of technology would have a negative impact on their well-being with no educational value (49). Furthermore, wearing Fitbits also made adolescents less motivated to engage in physical activity, with lower autonomous forms of motivation (50). In essence, the one crucial barrier to the acceptability of the Movement Index is the potential need for a wearable to inform the Movement Index. The need for a wearable raises some complexities in terms of potential access, engagement, and ethics. This issue will need to be resolved in future development of the Movement Index, while also considering alternative methods for collecting information on the movement behaviours of the child such as through parent self-report.

### 4.2 Limitations

Several limitations of this study should be acknowledged. The prototype did not fully represent the actual Movement Index which has not yet been created, and this artificial context may have influenced participants' understanding and interpretation of the Movement Index. Furthermore, the current study consists of participants who are mostly well-educated and former physical activity app users who may have been more predisposed to health and fitness. Caution is required when considering the transferability of the findings to other populations (33). However, it should be noted that most Canadians have a smartphone, and using health apps is quite common (18). Thus, the current sample may not be much different from the general Canadian population in terms of smartphone and health app use. Continued exploration of acceptability will be required as the Movement Index evolves and more diverse sampling will be required.

### 4.3 Future directions

The Movement Index is still in development, and the current study provides clear recommendations in moving forward to a usable prototype (see Table 3). After the Movement Index has been created, examining its acceptability at other time points will be important. The current project was situated at a pre-intervention time point, where prospective acceptability was explored to highlight whether one should proceed with this concept and, if so, what modifications might be needed to maximize its acceptability (25). Future research can examine the acceptability during or post intervention using prospective or retrospective temporal perspectives. An evaluation of usability, including the functionality and interface of the app, is another area that should be examined to ensure users can engage with the app effectively (51). A final long-term step would be assessing whether the app effectively supports children to engage in a healthy balance of movement behaviours.

## 5 Conclusion

The evaluation of the seven TFA constructs suggested that, on balance, the Movement Index is acceptable, with caveats that could be addressed in the future development of the Movement Index. The need for wearable tracking devices to inform the Movement Index raised concerns regarding accessibility and ethical implications. Thus, some type of manual data entry would be needed as an alternative option. With manual data entry, perhaps the Movement Index will be suitable as a short-term intervention to avoid burden and time/effort cost, such as an assessment tool to be used over 24 h once a week, an educational tool in classrooms to teach children about the 24HMG, or as a conversation starter between parents and children on optimally balancing movement behaviours. Overall, this study suggests proceeding with developing the Movement Index. Future development work is required to develop the Movement Index before examining its acceptability and usability as a knowledge translation tool to increase awareness of the guidelines and support parents and children in engaging in a healthier balance of movement behaviours.

## Data Availability

The raw data supporting the conclusions of this article will be made available by the authors, without undue reservation.
