# Supplementary material for: Development of a 24-hour movement index: exploring acceptability among Canadian parents
Source: Front Sports Act Living. 2025 Jul 30;7:1571207. doi: 10.3389/fspor.2025.1571207 (PMC12344945; doi:10.3389/fspor.2025.1571207)
Supplement: Supplementary file 1 [file Table1.docx]

Supplementary Material S1 – Interview Guide

**Guidelines Awareness Questions**

1. Have you ever heard of the 24-hour movement behavior guidelines?
2. If yes, where did you hear or read about them?
3. Can you recall any specific recommendation within the guidelines?

(e.g., amount of physical activity per day; amount of sleep per night)

**Guidelines Knowledge and Understanding Questions**

The following are the 24-Hour Movement Behavior Guidelines for Youth between the age of 5 to 17 (show slide):

- *Sweat: An accumulation of at least 60 minutes per day of moderate to vigorous physical activity involving a variety of aerobic activities. Vigorous physical activities, and muscle and bone strengthening activities should each be incorporated at least 3 days per week*
- *Step: Several hours of a variety of structured and unstructured light physical activities.*
- *Sleep: Uninterrupted 9 to 11 hours of sleep per night for those aged 5–13 years and 8 to 10 hours per night for those aged 14–17 years, with consistent bed and wake-up times.*
- *Sedentary Behavior: No more than 2 hours per day of recreational screen time; Limited sitting for extended periods.*

1. Do you think that these guidelines are important? In what way?
2. Do you think these guidelines are helpful for you as a parent? In what way?
3. Do you think these guidelines are realistic for children to achieve?
4. What resources do you have, or might be interested in receiving, to help your child meet these guidelines?

**Movement Index Questions**

1. Would you be interested in monitoring your child’s movement behaviors (physical activity, sedentary behavior, sleep) for their overall health benefit?
2. What might be the benefits of monitoring? Any disadvantages?
3. What challenges could potentially arise when monitoring your children’s movement behaviors?
4. What are your opinions of the specific features of this prototype? [AFFECTIVE ATTITUDE]

- Does the teeter totter convey the notion of a Movement Index? Or is there another way of conveying the Movement Index? [PERCEIVED EFFECTIVENESS]
- How do you like the look of it? [AFFECTIVE ATTITUDE]
- Anything unclear? [INTERVENTION COHERENCE]

1. Overall, how do you feel about what you’ve seen so far? (Overwhelmed, helpful/not helpful, simple/complex) [AFFECTIVE ATTITUDE]
2. How do you feel about those two options – manual or device data entry? [AFFECTIVE ATTITUDE]
3. Would you like to see any of these screen shots again – Is any feature unclear or confusing to you? [INTERVENTION COHERENCE]
4. Do you have a clear understanding of the proposed Movement Index and how it works? [INTERVENTION COHERENCE]
5. Overall, what do you think would be some advantages and disadvantages of using the Movement Index? [PERCEIVED EFFECTIVENESS]

Prompt: does it look easy or difficult to use? [BURDEN]

1. Are there any changes you would recommend in what you’ve seen? [AFFECTIVE ATTITUDE]
2. If a movement index like the one that is shown is created to monitor your children’s overall movement behavior, would it be something that you would use? why/why not? [INTENTION TO USE]
3. Would you have to give up anything in order to engage and use this app? [OPPORTUNITY COST]
4. Is it something that you see yourself using regularly? [INTENTION TO USE]
5. How well do you think the app can achieve its purpose? [PERCEIVED EFFECTIVENESS]
   1. help children progress towards the 24-hour movement guidelines
   2. knowledge translation of guidelines
   3. show the balance of movement behaviors
6. Are you confident that you can navigate through the app and use it appropriately and efficiently? [SELF EFFICACY]

**Closing Questions**

1. What is your overall opinion of the concept of the Movement Index, and this app as a knowledge translation tool of the guidelines? [AFFECTIVE ATTITUDE]
2. Does the movement index, and what it is trying to achieve, fit with your individual value system? Would it be helpful for all Canadians or would it have any exclusions? Does privacy of data concern you? [ETHICALITY]
3. What other methods of knowledge translation tools can you think of that can facilitate the translation of the 24-hour movement guidelines and convey the idea of balance between behaviors? (MESSENGERS)
4. In terms of promoting the movement index, from whom would you like to receive information the Movement Index? (METHODS)
5. How would you like to receive this information?

- Prompts: For example, in elementary school health classes, online through social media, etc.

1. Do you have any questions or comments? Anything else you would like to add?
